# MRI/PET multimodal imaging of the innate immune response in skeletal muscle and draining lymph node post vaccination in rats

**DOI:** 10.3389/fimmu.2022.1081156

**Published:** 2023-01-11

**Authors:** Saaussan Madi, Fang Xie, Kamyar Farhangi, Chih-Yang Hsu, Shih-Hsun Cheng, Tolulope Aweda, Bhasker Radaram, Stephanie Slania, Tammy Lambert, Mary Rambo, Tina Skedzielewski, Austin Cole, Valeriia Sherina, Shannon McKearnan, Hoang Tran, Hasan Alsaid, Minh Doan, Alan H. Stokes, Derek T. O’Hagan, Giulietta Maruggi, Sylvie Bertholet, Stéphane T. Temmerman, Russell Johnson, Beat M. Jucker

**Affiliations:** ^1^ Bioimaging, GSK, Collegeville, PA, United States; ^2^ Non Clinical Safety, GSK, Collegeville, PA, United States; ^3^ Research Statistics, GSK, Collegeville, PA, United States; ^4^ Vaccines Research & Development, GSK, Rockville, MD, United States; ^5^ Vaccines Research & Development, GSK, Rixensart, Belgium; ^6^ Clinical Imaging, GSK, Collegeville, PA, United States

**Keywords:** innate immune activation, magnetic resonance imaging, positron emission tomography, self-amplifying mRNA, lipid nanoparticle, AS01

## Abstract

The goal of this study was to utilize a multimodal magnetic resonance imaging (MRI) and positron emission tomography (PET) imaging approach to assess the local innate immune response in skeletal muscle and draining lymph node following vaccination in rats using two different vaccine platforms (AS01 adjuvanted protein and lipid nanoparticle (LNP) encapsulated Self-Amplifying mRNA (SAM)). MRI and ^18^FDG PET imaging were performed temporally at baseline, 4, 24, 48, and 72 hr post Prime and Prime-Boost vaccination in hindlimb with Cytomegalovirus (CMV) gB and pentamer proteins formulated with AS01, LNP encapsulated CMV gB protein-encoding SAM (CMV SAM), AS01 or with LNP carrier controls. Both CMV AS01 and CMV SAM resulted in a rapid MRI and PET signal enhancement in hindlimb muscles and draining popliteal lymph node reflecting innate and possibly adaptive immune response. MRI signal enhancement and total ^18^FDG uptake observed in the hindlimb was greater in the CMV SAM vs CMV AS01 group (↑2.3 – 4.3-fold in AUC) and the MRI signal enhancement peak and duration were temporally shifted right in the CMV SAM group following both Prime and Prime-Boost administration. While cytokine profiles were similar among groups, there was good temporal correlation only between IL-6, IL-13, and MRI/PET endpoints. Imaging mass cytometry was performed on lymph node sections at 72 hr post Prime and Prime-Boost vaccination to characterize the innate and adaptive immune cell signatures. Cell proximity analysis indicated that each follicular dendritic cell interacted with more follicular B cells in the CMV AS01 than in the CMV SAM group, supporting the stronger humoral immune response observed in the CMV AS01 group. A strong correlation between lymph node MRI T2 value and nearest-neighbor analysis of follicular dendritic cell and follicular B cells was observed (r=0.808, P<0.01). These data suggest that spatiotemporal imaging data together with AI/ML approaches may help establish whether *in vivo* imaging biomarkers can predict local and systemic immune responses following vaccination.

## Introduction

With an appreciation for the increased speed exhibited in developing Covid-19 mRNA vaccines, there is a need for scientific tools that will better establish differentiation of novel vaccine platforms while decreasing development time. The next generation mRNA and Adjuvant System (AS) based vaccine platforms will need imaging tools to better understand mechanism of action, to enable technology differentiation, and to effectively translate from cell to human. Key insights on how these vaccine platforms perform will enable future generations of vaccines to be developed with better performance characteristics (1).

Clinical scoring of reactogenicity has typically been evaluated as a compilation of (i) solicited events which include administration site pain, redness, and swelling and (ii) systemic events such as fatigue, fever, nausea, vomiting, diarrhea, abdominal pain, headache, myalgia, arthralgia, etc. These systemic events are classified as mild (Grade 1), moderate (Grade 2), severe (Grade 3), or potentially life threatening (Grade 4) (2). While these readouts have been useful as surrogates for acute innate immune response, there has nevertheless been a high incidence of reported adverse events when administered a placebo in clinical trials (3, 4). Therefore, there is a need for newer methods to temporally assess, quantify, and characterize the immune response, especially with respect to reactogenicity. Imaging approaches have long been used to examine the immune response following vaccination either at the site of injection, usually skeletal muscle, or in the draining lymph node (5–9). More recently, a larger industry-academic led initiative (ADITEC & BIOVACSAFE) has shown the utility of systems vaccinology and other high throughput, precision technologies for developing preclinical and clinical biomarkers of vaccine safety and reactogenicity (9, 10). This approach included the application of temporal fluorodeoxyglucose (^18^FDG) positron emission tomography (PET) imaging of injection site and draining lymph node immune response in healthy individuals receiving different vaccine/adjuvant combinations (9). ^18^FDG is the most commonly used tracer for PET imaging and has been used to assess various inflammatory conditions such as atherosclerosis, IBD, infection, vasculitis, etc. While it is non-specific, ^18^FDG tracer accumulates in metabolically activated innate/adaptive immune cells which are highly glycolytic, such as monocyte/macrophages and both T- and B-lymphocytes (11, 12). ^18^FDG PET provided excellent signal differentiation from background in muscle and draining lymph node following vaccination and importantly, the quantitative readouts were less variable than the standard clinical scoring of reactogenicity (9). Additionally, T2 weighted magnetic resonance imaging (MRI) is a sensitive biomarker for assessing musculoskeletal tissue inflammation and edema (13) and has been used in rodents and humans to characterize the acute temporal drug disposition and inflammatory response of a long acting injectable in muscle (14, 15).

Vaccine adjuvants have a central role in inducing transient inflammation at the delivery site that promotes immune cell recruitment and activation. This inflammation likely leads to better vaccine antigen uptake by critical-infiltrating cell types and migration of vaccine-loaded cells to the draining lymph nodes to establish adaptive immunity (16). While adjuvanted vaccines have been used safely in the clinic for many decades, Self-Amplifying mRNA (SAM) vaccines have only recently been tested in clinical trials (17). Therefore, the intent of the current study was to (1) combine PET and MRI approaches to non-invasively assess, in rats, the innate immune response elicited by SAM and AS (AS01) vaccine platforms using CMV mRNA (gB) or CMV recombinant protein (gB and pentamer), respectively, and (2) compare these *in vivo* imaging readouts to cytokine, antibody, and lymph node immune cell profiles. We assessed both skeletal muscle and lymph node imaging endpoints as well as cytokine and antibody responses temporally to capture the immune response to both Prime and Prime-Boost vaccination. Establishing *in vivo* imaging biomarkers of immune activation at injection site and draining lymph node will allow for non-invasive assessment of dynamic and temporal responses to currently used adjuvants with known transient reactogenicity (18) in vaccines and to benchmark against newer mRNA based lipid nanoparticle vaccine platforms both preclinically and clinically.

## Materials and methods

### Animal preparation and dosing

All animal procedures complied with the guidelines of the Institutional Animal Care and Use Committee at GSK following the guidance of Animal Use. All experiments were performed in male Sprague Dawley ((Crl: CD) SD) rats (Charles River Laboratories). Rats (250-300 g) were allowed food and water ad libitum and were acclimated for a minimum of one week prior to starting the study. Cytomegalovirus (CMV) antigen was used in the present study as there was availability of gB both as adjuvanted protein and as SAM construct. Two cohorts (Prime, and Prime-Boost) of 12 rats each received 50 μl injections, in the right gastrocnemius muscle, of one of the following:

- Adjuvanted vaccine; CMV AS01 (CMV: 10 μg gB + 20 μg pentamer), n=4/cohort.
- AS01 (5 μg QS-21 (*Quillaja saponaria* Molina, fraction 21; licensed by GSK from Antigenics LLC, a wholly owned subsidiary of Agenus Inc., a Delaware, USA corporation) + 5 μg MPL (3-*O*-desacyl-4′-monophosphoryl lipid A; produced by GSK) in liposomal formulation), n=2/cohort.
- Self-Amplifying mRNA vaccine; CMV SAM (CMV SAM: 10 μg CMV gB encoding mRNA encapsulated in LNP), n=4/cohort.
- LNP (empty lipid nanoparticle), n=2/cohort.

**Figure 1:**
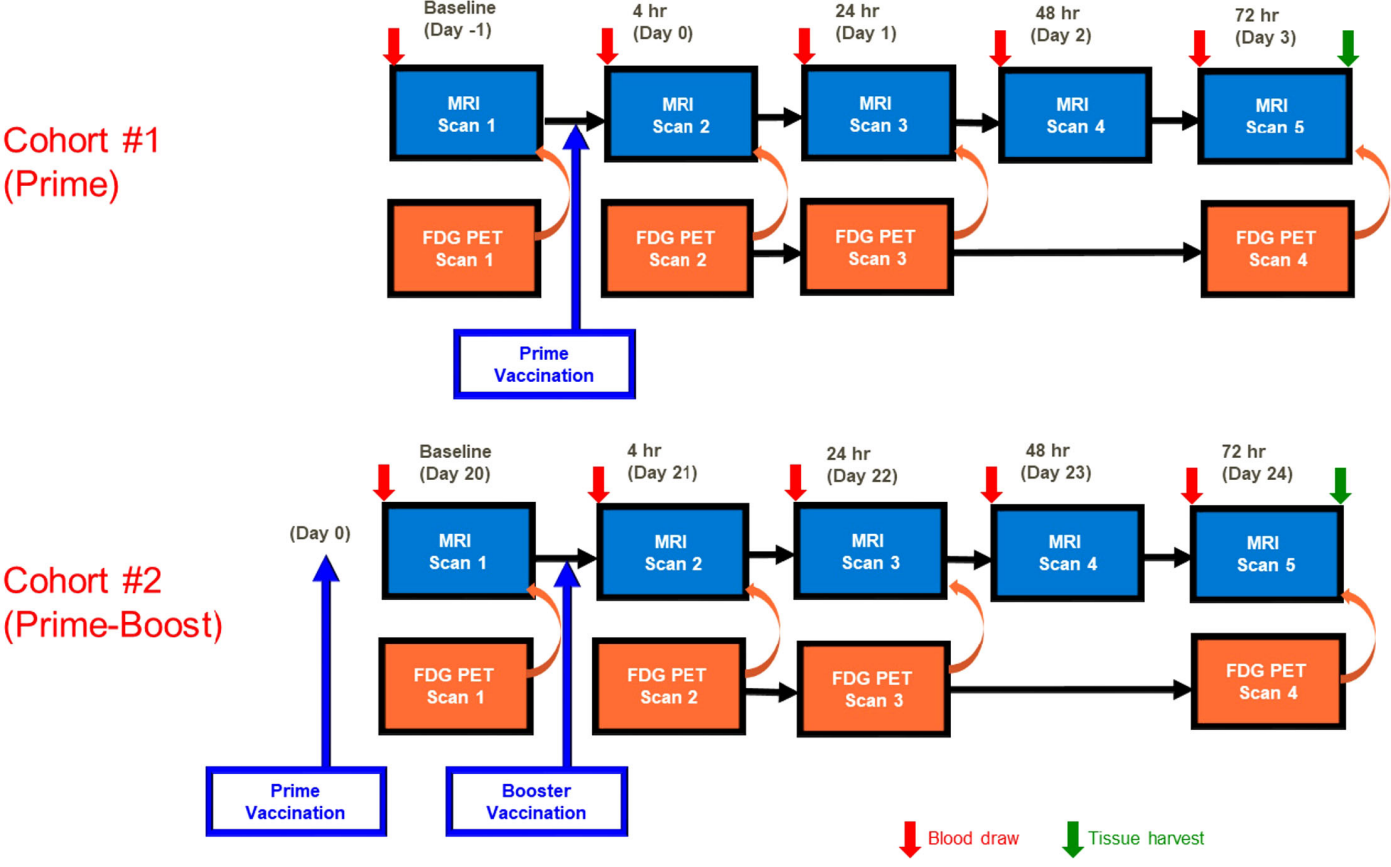
MRI and PET imaging timeline. MRI was performed at baseline (Day -1) and at 4 hr (D0), 24 hr (Day 1), 48 hr (Day 2), and 72 hr (Day 3) post Prime vaccination and at Days 20-24 (baseline through 72 hr post booster vaccination). ^18^FDG PET imaging was performed at all timepoints immediately prior to MRI with the exception of 48 hr post vaccination. Blood was collected prior to each imaging session, and gastrocnemius and popliteal lymph nodes were collected following the final imaging session.

The SAM vaccine formulations was prepared in a similar manner as previously described using a different antigen (19). Cohort 1 (Prime) was imaged following the Prime vaccine injection (Day 0). Cohort 2 (Prime-Boost) received a booster vaccine injection (Day 21) and was imaged only after receiving the booster vaccine injection. The MRI and PET imaging was performed at similar time points after the final vaccine injections, as shown in **Figure 1**.

### PET and MR imaging

Combined T2 (transverse relaxation rate) weighted MRI and ^18^FDG PET imaging of the right hindlimb muscles, draining right lymph node (popliteal), and spleen was performed at baseline (Day -1, Day 20) and at 4 hr (Day 0, Day 21), 24 hr (Day 1, Day 22), 48 hr (Day 2, Day 23), and 72 hr (Day 3, Day 24) post Prime or post Prime-Boost vaccine injection respectively in order to capture the early innate immune response (**Figure 1**). 200 μl of blood was obtained at baseline and prior to administration of ^18^FDG PET tracer at each imaging time point to measure plasma cytokines.

Serial ^18^FDG PET imaging using a Mediso LFER150 PET scanner (Mediso, Hungary) was followed by MRI performed on a 4.7T Bruker scanner (Bruker Biospin GmbH, Germany) using the same animal bed. Rats were fasted for a minimum of 4 hr prior to PET imaging to reduce basal ^18^FDG uptake, which occurs in muscle of non-fasted animals. PET scans were performed on anaesthetized (isoflurane 0.5-2%) rats 60 min following a tail vein administration of ~400 μCi ^18^FDG injection, so that tracer uptake in desired locations (e.g. vaccine injection site and draining lymph nodes) was optimized for PET imaging within ~60 min. A heating system was used to maintain animal body temperature at 37 °C. During the imaging session, the animal’s respiration was monitored using the integrated physiological monitor. Respiration rate and anaesthesia level were documented every 15 min. A CT scan (80 kV, 500 μA, 4 binning factor, 720 projections) was performed in each animal (for coregistration with the PET data and for CT-based PET attenuation correction scan) followed by a 15 min PET scan (same bed position). The acquired scan data was reconstructed using Tera-Tomo 3D reconstruction algorithm; 4 iterations; 8 subsets; 400-600 keV; 0.66 mm^3^ voxel size. The volume and the activity of each ^18^FDG dose was measured before injection, and the remaining dose in the syringe was measured to precisely calculate the injected dose. The acquired PET images were decay corrected to the injection time for each animal. Following the PET scan, the animal bed was moved to the MRI scanner with the animal remaining immobilized and under anaesthesia.

T2 weighted MRI was performed in order to assess local inflammation which results in a greater T2 value. The magnet was equipped with an 11.6 cm diameter actively shielded gradient set (660 mT/m). A birdcage volume coil (Bruker Biospin GmbH) with an inner diameter of 86 mm was used to obtain optimal radiofrequency homogeneity over the volume of interest. Anaesthesia was maintained with continuously inhaled isoflurane (0.5–3%) and a constant body temperature of 37 °C was maintained using warm air circulated over the body. Respiration was monitored using a respiratory sensor (SA Instruments, Inc., Stony Brook, NY) placed on the abdomen of the animal. Three scans were acquired: (1) a T2 weighted Rapid Acquisition Relaxation Enhancement (RARE) coronal scan with fat suppression, TR/TE = 3000/6.5 ms, RARE Factor = 16, TE_effective_ = 52 ms, FOV = 6 X 6 cm, matrix = 256 X 256, slice thickness = 1.5 mm, 25 slices, NEX = 8, and acquisition time of 4 min 48 sec. (2) T2 was measured with a coronal Multi-Slice Multi-Echo (MSME) sequence with fat suppression, TR/TE = 2500/7 ms, with number of echoes = 8 and the same geometry parameters as the RARE scan. (3) An axial scan with similar acquisition parameters to coronal MSME was acquired for lymph node volume and T2 measurements.

### Image analysis

PET/CT Image analysis was performed using VivoQuant 2021 software (InviCRO, Boston, MA) for the vaccine injection site, draining and non-draining lymph nodes, and spleen. Features were defined on the CT image to allow for the coregistration of the PET image. The volume of ^18^FDG increased uptake above background in the right hindlimb muscles was calculated as the volume of ^18^FDG signal that was 2 standard deviations (SD) above the mean ^18^FDG signal at baseline. Quantitative PET ^18^FDG uptake data were presented as a percentage of injected dose per gram of tissue (%ID/g). Total ^18^FDG uptake was calculated as %ID/g multiplied by the volume of increased ^18^FDG uptake (enhancement volume) in the right hindlimb muscles.

MRI image analysis was performed using Jim8 (Xinapse Systems Ltd, UK) software. The T2-weighted RARE scan was used to identify the volume of signal enhancement following vaccine injection. A region of interest (ROI) (greater than 2 x 2 in plane voxels) was placed on the left hindlimb (excluding the medial lipid regions, bones, muscle facial planes) as a control. The mean signal intensity + 2 SD in the left hindlimb was used as a lower threshold to identify the activation region in the right hindlimb. The volume of voxels exceeding this threshold were summed to obtain a signal enhancement volume. The T2-RARE ROIs were imported to T2 calculated parameter images from the coronal MSME scan. These ROIs were used to calculate the mean T2 from the coronal MSME image in the right hindlimb muscles. The calculated M0 parameter map on the axial MSME images was used to identify and draw ROIs to calculate volume of the popliteal lymph nodes in both hindlimbs. The ROI was copied to the axial T2 parameter images, and mean T2 was calculated for the popliteal lymph nodes.

Additionally, radiomics analysis was performed to find correlated imaging features with plasma cytokines and antibody titers. Radiomics features were computed using pyradiomics (20) for four imaging sequences (T2 and M0 MRI, PET, CT) with the ROIs annotated in the image analysis section. A total of 1400 radiomics features, including intensity, shape and texture features were computed for each sample. Univariate feature selection using correlation threshold was performed.

### Tissue and plasma analysis

After the final imaging sessions on Day 3 and Day 24, rats were euthanized. The gastrocnemius skeletal muscles and popliteal draining lymph nodes were harvested, and formalin fixed for immunohistochemistry (IHC) and Imaging Mass Cytometry (IMC).

Plasma was collected for cytokine analysis at baseline and prior to each imaging session. Cytokines were measured with the V-PLEX Plus Proinflammatory Panel 2 (rat) Biomarker Multiplex Assay (MesoScaleDiscovery, Gaithersburg, Md, Cat. No. KL5059G) according to the manufacturer’s instructions. Plates were analyzed on a Meso Scale Discovery Sector S 600. A final serum sample was collected from rats vaccinated with either platform (AS01 and SAM) for CMV gB antibody titer assessment in the Prime-Boost cohort. As a result of the final serum sample being collected 72 hr post dose, antibody response was forming, but not at peak titer.

### IMC analysis

Right popliteal lymph node samples collected at 72 hr post final vaccine injection were formalin fixed and paraffin embedded, and then sectioned at 4 µm thickness. The sections were analyzed by imaging mass cytometry on a Hyperion Imaging System (Fluidigm, South San Francisco, CA) after undergoing staining with metal-labelled monoclonal antibodies (21). On each lymph node section, a number of ROIs were selected to cover the entire lymph node. The dimension for each ROI was no larger than 1 mm x 1 mm. Antibodies were obtained in carrier-free buffer and then labelled using the MaxPar antibody conjugation kit (Fluidigm). Antibodies used in this study are listed in Supplementary Data (**Supplemental Table S1**). Heat-induced antigen retrieval was optimized to be 95°C in an EDTA-based pH 9 buffer for 1 hr. The Hyperion instrument settings were optimized as previously reported (21), and the imaging pixel diameter was fixed at 1 µm. The CyTOF software version 7 was used for data acquisition.

IMC data were analyzed using an in-house developed analysis pipeline. Briefly, cell segmentation was first performed on the images using machine learning-based pixel classification *via* Ilastik (22), and propagation of the nuclei pixels to membrane pixels followed by generation of the cell segmentation mask using CellProfiler (23). A cell segmentation mask was then used towards reading single-cell level average pixel intensities as surrogates for protein expression. Single cell expression data were then z-normalized and fitted by a 2-component Gaussian Mixture model to automatically recognize positively expressing from none-expressing cells at every single channel. Normalized and cleaned single cell protein expression data then went through a K-means clustering algorithm classifying cells into cell clusters with unique protein expression footprint. Each protein expression footprint was then visualized and assigned to a cell type/phenotype name based on the expression of their canonical markers. Cell type ratios were then calculated for each animal by aggregating the single cell information of all the imaged ROIs and calculating the ratio of every single recognized cell type/phenotype with respect to the overall cell population in the corresponding sample. The XY coordinates of each ROI were used to collate the mosaic whole section image of each sample and to perform the follow-up spatial relationship analysis globally.

Following the classification of cells into defined cell types, three global metrics were assessed. The cell type ratio was defined as the proportion of total cells that are designated as a particular cell type. The remaining two measures characterize the spatial relationship between two cell types, described here as cell type A and cell type B. The proximity between cell types, a measure of the quantity of nearby cells, was defined as the average number of cells of type B that each cell of type A is in close proximity to (i.e., 15 or less microns away). The nearest neighbor distance, a measure of how close one nearby cell is, was defined as the median distance between the closest cell of type B to each cell of type A.

### Statistical analysis

To compare both *in vivo* imaging and plasma cytokine endpoints across groups, area under curve (AUC) and maximum signal for each endpoint (38 in total) were used to assess differences between the groups. The maximum signal for each treatment group was classified by the timepoint with the highest average response among samples. These subsets of the data were used to compare the “peaks” of each treatment’s average signal curve across all observed timepoints. Separate Analysis of Variance (ANOVA) models were fit with a log-transformation on the AUC values and maximum signal values for each endpoint and cohort, with treatment as the independent variable. Additional models were fit combining the Prime and Prime-Boost cohorts into a single model with treatment, cohort, and the treatment-cohort interaction as independent variables. All models were fit in the statistical software R, version 4.1.1 (R Foundation for Statistical Computing, Vienna, Austria). Dose group comparisons (CMV AS01 vs CMV SAM, AS01 vs LNP) were made between treatments within a given cohort for all endpoints, and within the same group across cohorts. In order to control the family-wise error rate and guard against false concluding statistical significance between groups, the contrasts included Tukey’s p-value adjustment for all possible pairwise comparisons (24), though only those previously mentioned were of interest and studied. Descriptive statistics and Pearson correlations for the data were generated using Prism 8.0 software (GraphPad, San Diego, CA).

To compare IMC metrics defined in the previous section across treatment groups, the outcomes were calculated for each rat and modelled with a fixed effect for treatment. Cell type ratios were modelled using a beta regression with logit link; a pseudo count of 10^-5^ was added to any ratios equal to zero. Proximities between cell types were modelled using a linear regression with square-root transformed outcome. Nearest neighbor distances between pairs of cell types were modelled using a linear regression with log-transformed outcome. The treatment comparisons of interest were prespecified prior to data collection; thus, for all metrics, the p-values were unadjusted. The statistical analysis was performed using statistical software R, version 4.1.1 with the packages “glmmTMB” for beta regression, and “emmeans” for *post-hoc* comparisons and p-value calculations.

## Results

**Figure 2:**
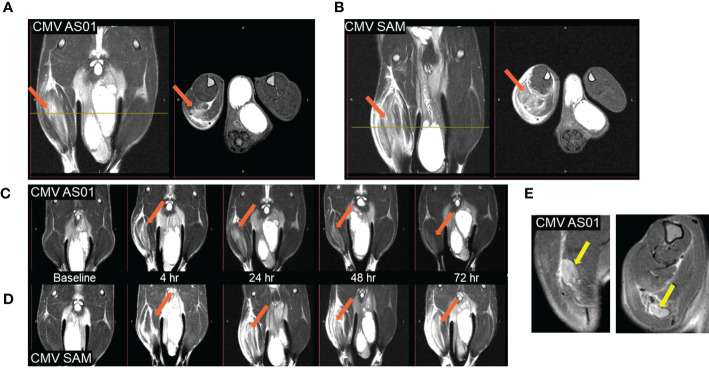
MR images of rat hindlimb at 24 hr post Prime vaccine injection. Coronal (left) and associated Axial (right) images (at level of yellow line slice indicator) for CMV AS01 **(A)** and CMV SAM **(B)**. Temporal response images for CMV AS01 **(C)** and CMV SAM **(D)**. Both Coronal (left) and Axial (right) images of the popliteal lymph node are identified **(E)**. Orange arrows indicate area of inflammation in hindlimb muscles following vaccination. Yellow arrows indicate popliteal lymph nodes.

**Figure 3:**
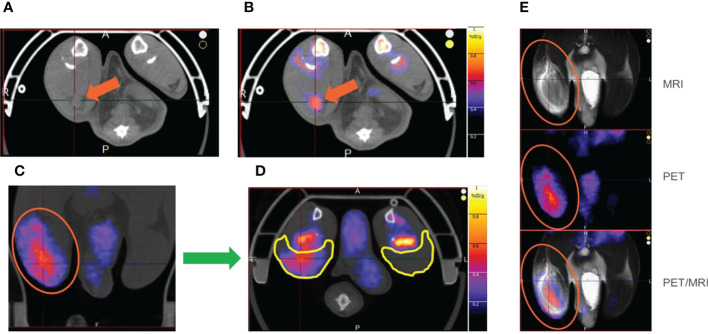
^18^FDG PET images of rat hindlimb at 24 hr post Prime vaccine injection. Axial CT image with right popliteal lymph node identified for CMV SAM **(A)** and with ^18^FDG PET image coregistered with the CT image **(B)**. Hindlimb ^18^FDG uptake in CMV SAM in Coronal **(C)** and Axial **(D)** planes. Coregistration of MRI and PET images **(E)**. Orange arrows indicate popliteal lymph node, and yellow ROIs indicate gastrocnemius muscle region.

T2 weighted MRI signal enhancement at the site of vaccine injection was used to assess innate immune activation (**Figure 2**). Following vaccine or control (adjuvant/LNP) injection into the right hindlimb (shown on left side in radiological oriented images) there was significant signal enhancement and the volume of signal enhancement increased with inflammation and associated edema. The greatest signal enhancement appeared between 4 and 24 hr time points. The signal enhancement volume in the right hindlimb muscles of the CMV SAM group appeared not only greater at 24 hr (**Figure 2A, B**), but longer in duration (**Figure 2C, D**) than in the CMV AS01 group. There was no signal enhancement observed in the contralateral hindlimb, suggesting the local nature of the induced early reaction. MRI captured the draining popliteal lymph node in images which were used to measure lymph node volume changes throughout the duration of the experiment (**Figure 2E**). Additionally, CT images were used to identify the popliteal lymph node and adjacent muscle beds for PET image coregistration (**Figure 3A**). ^18^FDG PET signal was detected in both right popliteal lymph node (**Figure 3B**) and right hindlimb muscles (**Figures 3C, D**) following vaccination. Similar to MRI, there was little to no uptake in contralateral gastrocnemius muscle (highlighted yellow ROIs) in **Figure 3D** or in contralateral popliteal lymph node (**Supplemental Figure S1**). The regions of MRI signal enhancement and ^18^FDG PET uptake in the right hindlimb were similar as can be observed in the PET/MRI coregistered image (**Figure 3E**). Additional baseline and 24 hr post Prime vaccination MR and ^18^FDG PET images for all dose groups are shown in **Supplemental Figure S1**.

**Figure 4:**
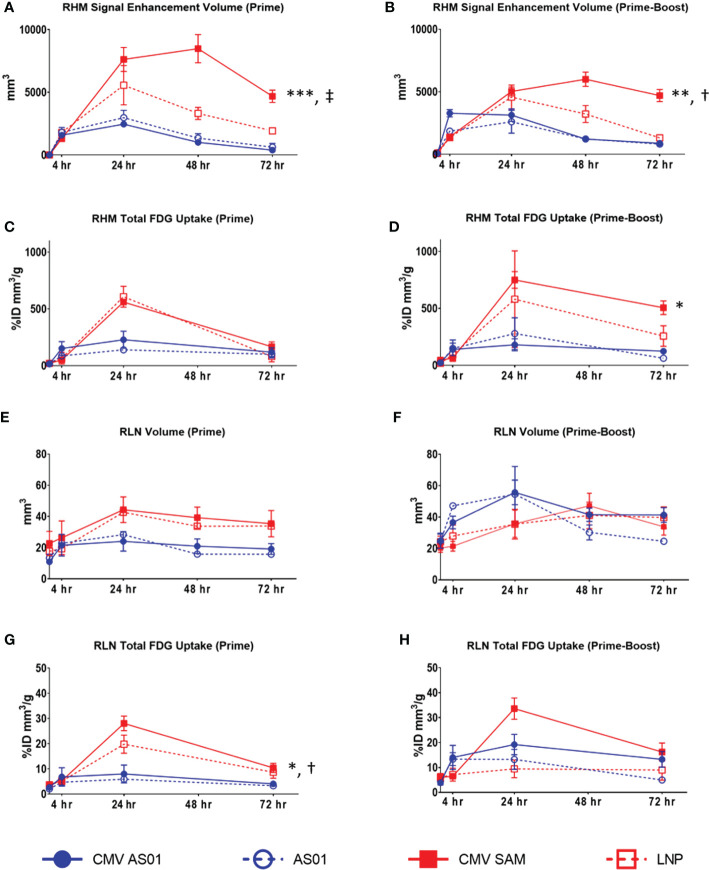
Quantitative MRI and PET data readouts in right hindlimb muscles and right popliteal lymph node. MRI enhanced signal volume in right hindlimb muscles (RHM) following Prime **(A)** or Prime-Boost **(B)** vaccine injection. ^18^FDG PET derived total ^18^FDG uptake in RHM following Prime **(C)** or Prime-Boost **(D)** vaccine injection. MRI measured right lymph node (RLN) volume following Prime **(E)** or Prime-Boost **(F)** vaccine injection. PET derived total ^18^FDG uptake in RLN following Prime **(G)** or Prime-Boost **(H)** vaccine injection. Data are presented as mean ± SEM. ^*^P<0.05 CMV SAM AUC vs CMV AS01 AUC; ^**^P<0.01 CMV SAM AUC vs CMV AS01 AUC; ^***^P<0.001 CMV SAM AUC vs CMV AS01 AUC; ^†^P<0.05 CMV SAM maximum vs CMV AS01 maximum; ^‡^P<0.001 CMV SAM maximum vs CMV AS01 maximum.

In the CMV AS01 Prime dose cohort, the MRI signal enhancement volume in the right hindlimb muscles increased as early as 4 hr and peaked at 24 hr before resolving back to baseline at approximately 72 hr (**Figure 4A**). However, the CMV SAM Prime dose cohort peaked at 48 hr and remained elevated at 72 hr (**Figure 4A**). The response following Prime-Boost vaccine administration was generally similar to that observed in the Prime dose cohort with the exception of CMV AS01 possibly peaking earlier at 4 hr (**Figure 4B**). The average maximum MRI signal enhancement volume was 8481 mm^3^ in the CMV SAM group as compared to 2458 mm^3^ in the CMV AS01 group following Prime dose, and 6000 mm^3^ and 3283 mm^3^ in the CMV SAM and CMV AS01 groups respectively following Prime-Boost dose (P<0.05 between groups in both cohorts). Additionally, MRI signal enhancement volume AUC was calculated to be 4.3 and 2.3 fold greater in the CMV SAM vs CMV AS01 group following Prime and Prime-Boost vaccine injection respectively (P<0.01). Similar temporal responses were observed in the AS01 or LNP groups within respective dose cohorts. However, the CMV SAM response appeared to be more persistent than LNP alone. Additionally, we tested the hypothesis that increased interstitial pressure from needle injection or dose volume would drive the observed inflammatory response. This was done by performing a saline infusion in a separate cohort of animals. The maximum signal enhancement volume following saline administration was only 88 mm^3^ at 4 hr and resolved by 24 hr (data not shown). T2 in right hindlimb muscles increased as early as 4 hr in both Prime and Prime-Boost cohorts and peaked at approximately 24 hr. T2 AUC was 1.3 and 1.2 fold greater in CMV SAM vs CMV AS01 group in both Prime and Prime-Boost cohorts respectively (P<0.01, **Supplemental Figure S2**). While total ^18^FDG uptake in right hindlimb muscles following ^18^FDG PET reflected a similar response profile to MRI following both Prime and Prime-Boost administration, there was a significant difference in total ^18^FDG uptake AUC in CMV SAM vs CMV AS01 group in the Prime-Boost cohort (↑3.9 fold, P<0.05, **Figures 4C, D**). Total glycolytic burden in the right hindlimb muscles was associated mainly with increased volume of ^18^FDG uptake (**Supplemental Figure S3**). Although not significant, MRI-derived right popliteal lymph node volume AUC appeared to be greater in CMV SAM vs CMV AS01 group in the Prime dose cohort (**Figure 4E**) and AS01 administration appeared to increase right popliteal lymph node volume in the Prime-Boost cohort (**Figure 4F**). There were no statistically significant differences in right popliteal lymph node T2 between dose groups in both Prime and Prime-Boost cohorts (**Supplemental Figure S2**). Total ^18^FDG uptake in the right popliteal lymph node was higher in CMV SAM vs CMV AS01 group in the Prime cohort (↑3.5 fold AUC, ↑4.5 fold Maximum, P<0.05), mainly driven by the increased ^18^FDG uptake in the lymph nodes (**Figures 4G, H**, **Supplemental Figure S3**). While total ^18^FDG uptake remained generally low throughout the experiment in contralateral hindlimb muscles and popliteal lymph node (<50%ID mm^3^/g and ~2-6%ID mm^3^/g respectively in all groups and cohorts) there was an appreciable increase in CMV AS01 contralateral hindlimb muscles at 72 hr post dose (~100%ID mm^3^/g) in both cohorts (data not shown). Additionally, there was a slight increase ^18^FDG uptake in spleen observed in CMV AS01 Prime vs Prime-Boost cohort over the observed timeframe (↑1.4 fold AUC, P<0.05, **Supplemental Figure S4**).

**Figure 5:**
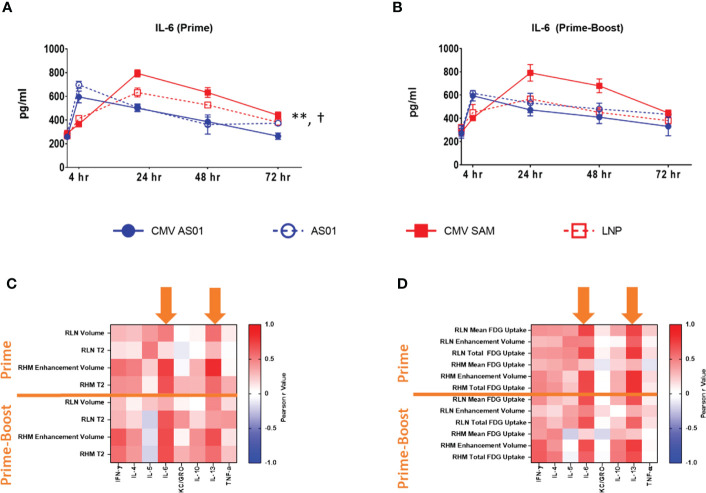
Cytokine correlation with MRI and PET imaging endpoints. Plasma IL-6 temporal response following Prime **(A)** and Prime-Boost **(B)** vaccine injection. Heat map of Pearson correlations between plasma cytokine/chemokines and MRI **(C)** and ^18^FDG PET **(D)** endpoints in right hindlimb muscles (RHM) and right lymph node (RLN) following Prime and Prime-Boost vaccine injection. Orange arrows indicate strong correlations between IL-6 and IL-13 with MRI and ^18^FDG PET endpoints. Data are presented as mean ± SEM. ^**^P<0.01 CMV SAM AUC vs CMV AS01 AUC; ^†^P<0.01 CMV SAM maximum vs CMV AS01 maximum.

A panel of pro-inflammatory cytokines were assessed in plasma taken prior to each imaging session. KC/GRO was maximally increased by 8.7 and 6.1 fold in CMV AS01 vs CMV SAM group in both Prime and Prime-Boost cohorts respectively (**Supplemental Figure S5**, P<0.001). While other cytokines did not exhibit such large dynamic changes, IL-4, IL-6 and IL-13 AUCs were increased in CMV SAM vs CMV AS01 group in the Prime cohort (P<0.05) and other cytokine AUCs (IL-10, TNF-α) were similarly elevated in CMV AS01 and CMV SAM groups in both Prime and Prime-Boost cohorts (**Figure 5A**, **Supplemental Figure S5**). IL-6 peak response was at 4 hr and 24 hr following CMV AS01 and CMV SAM administration respectively in the Prime cohort (P<0.05, **Figures 5A, B**). Correlation analysis was performed between cytokines and both MRI and PET imaging endpoints with IL-6 and IL-13 showing the strongest relationships (**Figures 5C, D**). In addition, we evaluated the relationship between MRI and PET endpoints to understand similarities and differences in order to better utilize these endpoints in future studies. We found that the two strongest correlations were in (1) the right hindlimb muscles, between MRI T2, and PET enhancement volume and total ^18^FDG uptake in both Prime and Prime-Boost administration cohorts, and in (2) the popliteal lymph node, where volume assessed by MRI and PET correlated, and MRI T2 strongly correlated with the ^18^FDG PET volume measurement in the Prime cohort. Additionally, there were other positive correlations between MRI and PET imaging measurements (**Supplemental Figure S6**).

**Figure 6:**
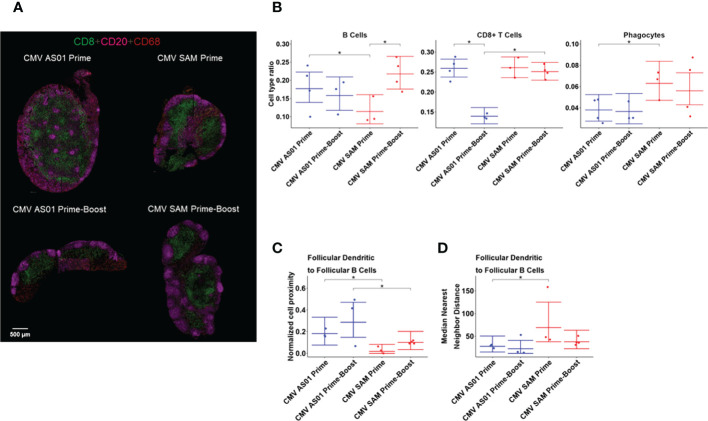
IMC images of popliteal lymph nodes and immune cell interaction analysis. Representative IMC images of CD8, CD20, and CD68 from CMV AS01 Prime, CMV SAM Prime, CMV AS01 Prime-Boost, and CMV SAM Prime-Boost lymph nodes **(A)**. The cell type ratio comparisons between treatments for three cell populations: B cells, CD8+ T cells, and Phagocytes **(B)**. The normalized cell proximity describes the average number of follicular B cells near to each follicular dendritic cell in a given rat **(C)**. The nearest neighbor distance is the distance between a follicular dendritic cell and the closest follicular B cell and is presented as the median of those distances, summarized per rat **(D)**. Data are presented as the mean and the 95% CI. *P<0.05 between respective groups.

Imaging mass cytometry was used to generate high content, broad immune cell phenotyping in the draining popliteal lymph node (**Figure 6A**). From the panel of markers imaged, a list of cell types was identified, including antigen presenting cells (MHCII+), B cells (CD20+), CD8+ T cells (CD3+CD8+), follicular B cells (CD20+Bcl6+), helper T cells (CD3+CD4+), phagocytes (CD68+), regulatory T cells (CD4+FoxP3+), follicular dendritic cells (CD21+CD20-), and follicular helper T cells (CD3+Bcl6+) (**Supplemental Figure S7, S8**). When the cell type ratio in the draining popliteal lymph node was compared between different treatments, the ratio of B cells was identified as being significantly higher in the CMV AS01 vs CMV SAM group in the Prime dose cohort (**Figure 6B**), associated with greater numbers of B cells under development in the draining lymph node at this time point in the CMV AS01 vs CMV SAM prime group. However, the booster administration significantly increased the B cell development in the draining lymph node in the CMV SAM group. The CD8+ T cell ratio in the draining lymph node was maintained for at least 72 hours after booster administration in the CMV SAM group while the ratio was reduced in the CMV AS01 group following booster administration. In contrast, CMV SAM treatment induced a significantly higher ratio of phagocytes than CMV AS01 treatment in the Prime cohort, reflecting a strong cellular immune response induced by CMV SAM injection (**Figure 6B**).

Cellular interactions were examined by performing cellular proximity and nearest- neighbor analysis. The proximity analysis indicated that on average, follicular dendritic cells interacted with more follicular B cells in the CMV AS01 treatment than in the CMV SAM treatment in both cohorts (**Figure 6C**). The nearest-neighbor analysis corroborated this result by showing a significantly lower median distance between the follicular dendritic cells and their nearest follicular B cells in the CMV AS01 treatment, than in the CMV SAM treatment in the Prime cohort (**Figure 6D**). We further examined the relationship between IMC cellular readouts in the vaccine treatment groups with MRI and PET endpoints and observed a number of strong associations, among which the strongest positive correlation was between right popliteal lymph node T2 and the nearest neighbor of follicular dendritic cells and follicular B cells (r=0.808, P<0.01). Additionally, a correlation between right hindlimb muscles MRI enhancement volume and nearest neighbor antigen presenting and helper T cells (r=0.704, P<0.01) was observed (**Supplemental Table S2**). The ratio of CD8+ T cells was negatively correlated with the CMV gB antibody titer in Prime-Boost groups at 72 hr post-booster administration (r=-0.918, P<0.01) indicating that humoral immunity and cellular immunity kinetics may be different between vaccine treatment groups.

**Figure 7:**
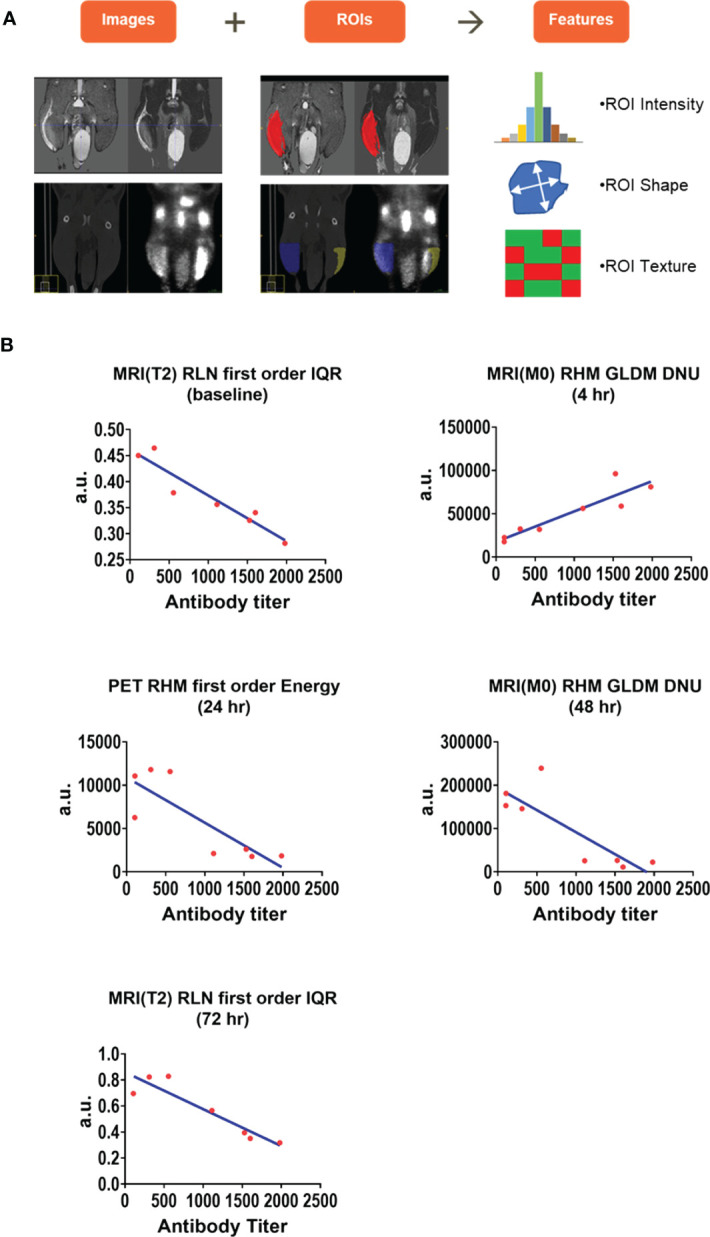
Radiomics analysis of right hindlimb and right draining lymph node images. Radiomics features were extracted from the Prime-Boost cohort images of hindlimb and draining lymph node to find predictors of immune response. 107 image features including region of interest (ROI) intensity, ROI shape, and ROI texture were extracted from each MRI, PET, and CT image of the right hindlimb and draining popliteal lymph node **(A)**. Hindlimb and draining lymph node features were correlated with biomarkers of immune response including CMV gB antibody titers at each timepoint and highly correlated features are presented **(B)**. a.u. (arbitrary units), IQR (interquartile range), GLDM DNU (Gray Level Dependence Matrix Dependence Non-Uniformity).

Radiomics analysis was performed on the Prime-Boost cohort MR, PET, and CT images to extract imaging features which were highly correlated with CMV gB antibody titers at all imaging timepoints. **Figure 7A** illustrates MR (top row) and PET/CT (bottom row) images with ROIs overlayed in red and blue for right hindlimb MRI and PET/CT respectively. Imaging features such as intensity, shape, and texture were extracted. **Figure 7B** shows the highest correlated radiomics features at each timepoint. Specifically, interquartile range of T2 intensity in the draining lymph node was highly correlated at baseline and 72 hr, Gray Level Dependence Matrix (GLDM) Dependence Non-Uniformity (DNU) of M0 in the hindlimb signal enhancement volume was highly correlated at 4 hr and 48 hr, and first order energy of PET signal intensity in the hindlimb was highly correlated at 24 hr (see Supplemental Materials for detailed description of these features). The time-dependent correlation suggests that T2 intensity features may indicate early and late effects of immune response. Correlation of M0 texture feature GLDM DNU of the hindlimb changed from highly positive at 4hr to highly negative at 48 hr showing this M0 texture feature was related to increasing immune response. Additionally, a first order energy feature of the PET signal intensity in the hindlimb may capture peak immune response.

**Figure 8:**
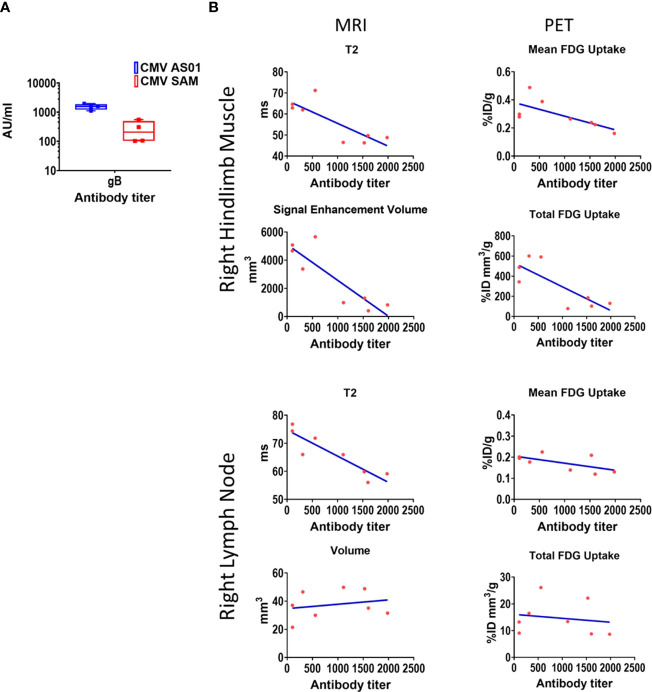
CMV antibody titers and correlation to MRI and PET imaging endpoints. Antibody titers for CMV gB (CMV AS01 and CMV SAM) were measured 72 hr post Prime-Boost vaccination **(A)**. Pearson correlations between CMV gB antibody titers and right hindlimb and draining lymph node MRI and ^18^FDG PET endpoints **(B)**. CMV gB antibody data are shown in box and whisker plot format. AU (arbitrary units).

While not designed for optimal response, CMV gB antibody titers were assessed in the Prime-Boost cohort only at termination of the imaging study (i.e., 72 hr). Nevertheless, titers were approximately 10 fold higher for gB in the CMV AS01 vs CMV SAM group (**Figure 8A**). Correlation analysis was performed between MRI and ^18^FDG endpoints and antibody response (**Figure 8B**). Negative correlations between MRI T2 values in both right hindlimb muscles and right popliteal lymph node and antibody titer were observed (P<0.05 and P<0.01 respectively). Additionally negative correlations in the right hindlimb muscles between MRI signal enhancement volume and antibody titer, and between PET total ^18^FDG uptake and antibody titer were observed (P<0.01 and P<0.05 respectively). However, the negative correlations observed were mainly driven by the large dose group differences in antibody titers.

## Discussion

Historically, the industry-wide development of new adjuvant systems has been slow, with only 4 new adjuvant systems marketed in the last 20 years (25). This is in part a result of insufficient mechanistic understanding of these novel adjuvant systems and of the interplay between immunogenicity and reactogenicity. Whole blood transcriptomics, circulating immune cell, or cytokine profiling are routinely used for identification of biomarkers of immunogenicity (26). However, routine biomarkers for predicting vaccine safety or reactogenicity are yet to be established (9, 10). Recent advancements include the use of systems vaccinology approaches to better understand systemic and local site reactogenicity (10, 18, 27). However, these approaches do not offer direct surveillance of the vaccine injection site and the proximal draining lymph node.

The present study represents the first application of a clinically translatable, combined MRI and PET imaging methodology for the simultaneous and non-invasive assessment of immune activation in muscle and draining lymph node following vaccination. Both CMV AS01 and CMV SAM resulted in rapid MRI and PET signal enhancement in hindlimb muscles and draining popliteal lymph node, reflecting innate and eventually adaptive immune modulation. Additionally, a number of *in vivo* imaging readouts in hindlimb muscles and popliteal lymph node correlated with systemic cytokine responses, lymph node immune cell profiles and germinal center interactions. These readouts enable more accurate predictions from pre-clinical models for likely reactogenicity/tolerability of next generation adjuvant and mRNA formulated vaccines.

To date there have been many clinical investigations assessing draining lymph node ^18^FDG uptake following vaccination in both retrospective and prospective PET imaging studies (5, 6, 9, 28–30). While there has been broad application of ^18^FDG PET imaging to assess immune response to vaccines in draining lymph nodes, there has been limited application to injection site imaging for reactogenicity assessment (9). The robust total ^18^FDG uptake response observed in the right hindlimb muscles (up to 25.4-fold above baseline) was accompanied in some animals by a decrease in ^18^FDG uptake in the adjacent highly glycolytic soleus muscle. This may reflect a metabolic steal to supply the increased metabolic needs of the activated innate immune cells in adjacent muscle. In addition, the increased hindlimb muscles MRI signal enhancement volumes and ^18^FDG uptake observed in the CMV SAM and LNP groups appeared to be driven in part by LNP uptake, possibly reflecting adjuvant characteristics of cationic lipids (31).

Trained immunity involves a host of metabolic and epigenetic modifications to innate cells and has recently been considered as a target to modulate with amplifier approaches in addition to traditional antigen/adjuvant combinations (32, 33). While cellular intermediary metabolism may be interrogated using other PET targeting or ^13^C hyperpolarized MRI approaches (34), ^18^FDG PET may nevertheless provide some insight into increased innate cellular metabolic demand reflecting innate trained immunity (35). Although speculative, AS01 administration alone resulted in an early increase popliteal lymph node volume and total ^18^FDG uptake measured in the Prime-Boost vs Prime cohort, possibly reflecting the contribution of metabolism to the adjuvant effect and to trained innate immunity. Additionally, mean ^18^FDG uptake measured in the draining popliteal lymph node correlated with phagocytes, possibly identifying an immune cell population that may be involved in trained innate immunity.

T2 weighted MRI has been used to assess drug depots in skeletal muscle following administration of long acting injectables, and signal increase associated with acute inflammatory response (15, 36). However, its application to vaccine immune response monitoring has been limited. Both absolute T2 and signal enhancement volume in the right hindlimb muscles were robustly increased 24 hr and 48 hr following CMV SAM administration in Prime and Prime-Boost cohorts. This is temporally similar to what has been observed following long acting injectable drug administration in muscle (14), although the response mechanisms are different. Robust dynamic responses were observed in MRI signal enhancement volume following both CMV AS01 and CMV SAM administration, but the absolute difference in these responses was intriguing. The increased response following CMV SAM administration suggests this readout as a sensitive biomarker for mechanism of reaction including local reactogenicity. Indeed, Bernau et al. have successfully used MRI to assess local reaction for a number of veterinary vaccines in pigs and sheep (37–40). Additionally, MRI was used by Brewer et al. and DeBay et al. separately to assess draining lymph node volume as a biomarker for successful vaccination treatment response in tumor bearing mice (41, 42). While we did not observe a correlation between MRI-assessed draining popliteal lymph node volume and antibody titers in the present study, this may be due to the small sample size and the sub-optimal timing of sample collection for maximal antibody production. The majority of MRI and PET imaging readouts correlated with each other. The strongest correlations were (1) right hindlimb muscles ^18^FDG PET enhancement volume and total ^18^FDG uptake with MRI T2, and (2) lymph node MRI volume and T2 with ^18^FDG PET enhancement volume. Right hindlimb muscles MRI signal enhancement and ^18^FDG PET total ^18^FDG uptake responses were similar indicating that either imaging biomarker may be appropriate. However, the^18^FDG PET uptake in lymph node provided a unique measure of immune activity to differentiate vaccine platforms that could not be achieved using MRI.

While systemic cytokines exhibited a similar temporal response to that of the *in vivo* imaging endpoints, the absolute cytokine responses were generally less robust than with imaging readouts. For example, in CMV AS01 and CMV SAM Prime cohorts, IL-6 maximally increased 1.3- and 1.7-fold vs baseline respectively, while PET-derived total ^18^FDG uptake increased 13.4- and 25.4-fold respectively at 24 hr. MRI signal enhancement was similarly robust due to the absence of detectable inflammation or edema at baseline. Additionally, maximum IL-6 response was only 33% greater in CMV SAM vs CMV AS01 Prime cohort while maximum MRI signal enhancement volume in the right hindlimb muscles was 245% greater. Similarly, the absolute dynamic range for PET-derived total ^18^FDG uptake in the right lymph node was greater than the cytokine responses albeit to a lesser extent than in the right hindlimb muscles. More recently, it was shown that the IL-1 receptor antagonist axis regulates vaccine-mediated systemic inflammation and is dependent on both RNA and lipid formulation (43). While we did not measure IL-1 in the current study, it does induce IL-6 modulation with greater dynamic range than IL-1. Therefore the *in vivo* imaging readouts of skeletal and/or draining lymph nodes may provide a more sensitive measure of local reactogenicity than systemic cytokine responses alone.

Although quantitative immune cell phenotyping in lymph node can be performed using flow cytometry methods, spatial cellular relationship analysis allows evaluation of cellular interactions in the lymph node anatomical/histological features including follicle and subcapsular regions. Proximity and nearest neighbor readouts (**Supplemental Figure S9, S10**) showed increased interactions between antigen presenting cells and helper T cells, and between follicular dendritic cells and follicular B cells in CMV AS01 vs CMV SAM group. These increases resulted in increased B cell numbers and humoral immunity. Additionally, in CMV SAM vs CMV AS01 group there was an increase in CD8+ T cells following Prime-Boost administration and an increase in phagocytes following Prime administration. While an increase in CD8+ T cells has been observed in splenocytes from previous studies with SAM constructs (19, 44), this is the first report of a CD8+ T cell increase in the draining lymph node. In addition, the increase in lymph node phagocytes proximal to follicular B cells in CMV AS01 vs CMV SAM Prime-Boost cohort (**Supplemental Figure S9**) is a unique finding that may underscore the role for phagocytes in robust immune stimulation and humoral response (45). Antigen presenting cells were also significantly lower in the CMV SAM Prime-Boost vs Prime cohort (**Supplemental Figure S7**) possibly highlighting a mechanism for reduced germinal center activity in the CMV SAM vs CMV AS01 group.

The relationship between the *in vivo* imaging endpoints (e.g., MRI signal enhancement, total ^18^FDG uptake, etc.) and IMC-derived cellular content or proximity readouts suggests an association between these imaging biomarkers with innate and adaptive immune responses in the lymph node. Strong relationships were observed between lymph node T2 and 1) antigen presenting cells, and 2) follicular dendritic cell and follicular B cell interactions, possibly reflecting a readout of adaptive immune response. While T2 may be increased with edema, there is less evidence that it is associated with a particular cell type or increase in cellularity. Additionally, MRI signal enhancement volume in the right hindlimb muscles appeared to be significantly correlated with lymph node phagocyte content and interaction between antigen presenting cells and helper T cells. While the immune cell environment wasn’t characterized in the gastrocnemius in this study, it can only be speculated that the increased signal observed was associated with the innate response of macrophages and antigen presenting cells at the vaccine injection site.

Future implementation of AI/ML-based radiomics may show how imaging features in the spatiotemporally derived MRI and PET data may be associated with both reactogenicity and immunogenicity biomarkers. This comprehensive approach might be used with systems vaccinology approaches to investigate whether these imaging biomarkers can help predict immune responses and cellular profiles at site of vaccine injection and draining lymph node (26). Future clinical experimental medicine imaging studies have the potential to translate the current study findings to support reactogenicity biomarker assessment in a similar manner to that used in the BIOVACSAFE and ADITEC consortia effort (9). This illustrated the advantage of quantitative imaging biomarkers in the muscle over the highly variable standard solicited reactogenicity surveys. Such imaging readouts could allow the use of fewer subjects in a systems vaccinology approach to optimizing formulations and limiting reactogenicity (10).

In conclusion, the present study established the utility of a multimodal *in vivo* imaging approach to temporally assess both vaccine injection site and draining lymph node immune activation. The study was exploratory in nature and was not designed for statistical analysis comparisons, or analytical fitting of the immune response, which may be a better approach for describing the immune response and comparing groups. Nevertheless, leveraging these standard imaging applications that are available in most research settings for both preclinical and clinical utility using both marketed AS (e.g., AS01, AS03) and/or novel vaccine platforms (e.g. SAM, mRNA) offers a unique opportunity to strengthen our knowledge of their mode of action. Besides the fundamental understanding of immune response that enables evidence-based development of future adjuvants and RNA based vaccines, imaging approaches can also open new avenues for combining with pharmacodynamic readout of vaccine efficacy. Extending the scope of work to other vaccine formulations, delivery platforms, or administration paradigms may also unveil novel mechanisms that may serve as asset differentiator for various vaccine platforms. Key insights on how these platforms perform together using a systems vaccinology approach will enable one to make the necessary antigen, adjuvant, and formulation modifications to build the next generation of vaccines with better performance characteristics. This non-invasive imaging assay highlights the utility of such an imaging readout for discovery applications and in the clinic.

## Data availability statement

The original contributions presented in the study are included in the article/**Supplementary Material**. Further inquiries can be directed to the corresponding author.

## Ethics statement

The animal study was reviewed and approved by GSK Institutional Animal Care and Use Committee.

## Author contributions

SM, FX, C-YH, DO’H, GM, SB, ST, RJ, and BJ contributed to conception and design of the study. SM, S-HC, TA, SS, MR, TS and BJ performed the in-life experiments. SM, KF, C-YH, SS, and TL organized the database. AC, VS, SM, and HT performed the statistical analysis. SM and BJ wrote the first draft of the manuscript. SM, FX, C-YH, AC, VS, and BJ wrote sections of the manuscript. All authors contributed to the article and approved the submitted version.
