# Supplementary material for: MRI/PET multimodal imaging of the innate immune response in skeletal muscle and draining lymph node post vaccination in rats
Source: Front Immunol. 2023 Jan 11;13:1081156. doi: 10.3389/fimmu.2022.1081156 (PMC9874296; doi:10.3389/fimmu.2022.1081156)
Supplement: Supplementary file 1 [file DataSheet_1.pdf]

Supplemental Materials:

Supplemental Figure S1

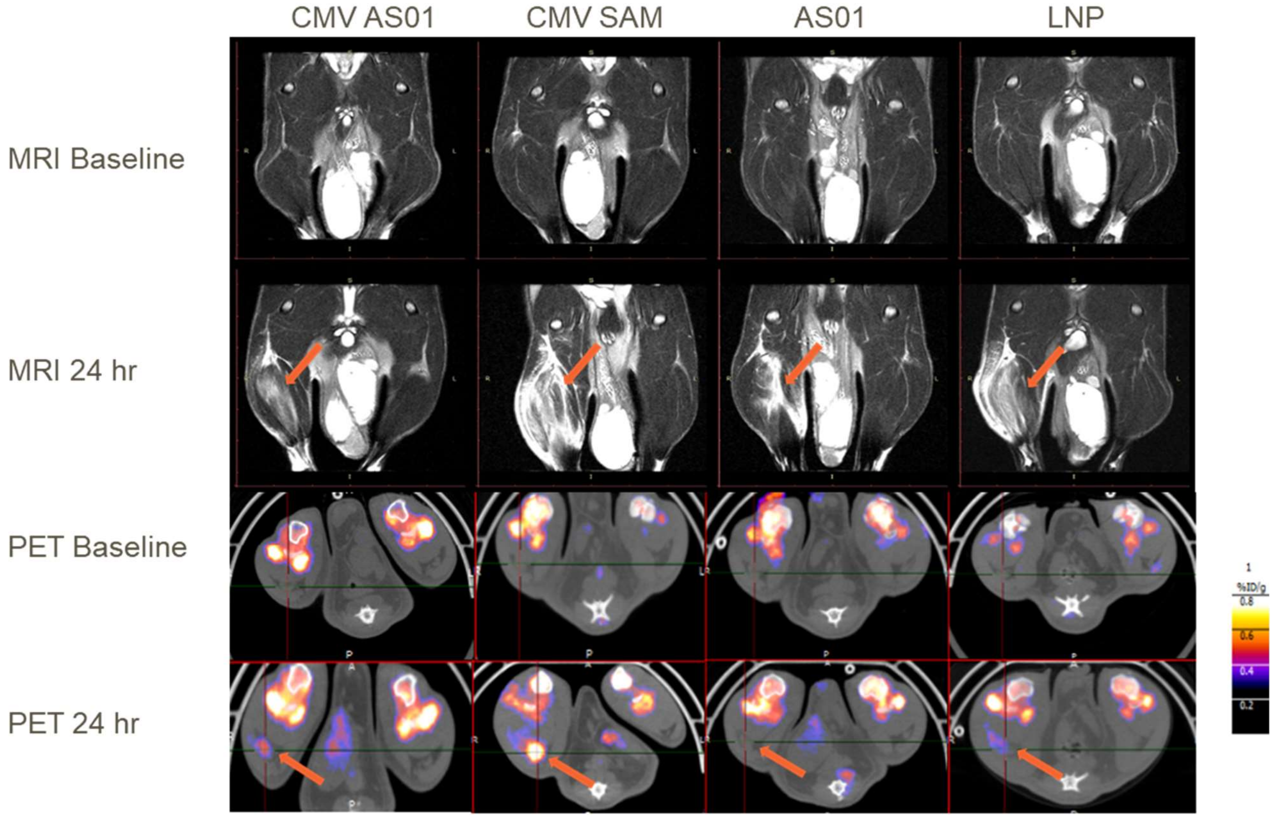

MR and PET images at baseline and 24 hr post Prime vaccine injection. T2 weighted coronal MR images of the hindlimbs for all dose groups at baseline and 24 hr post Prime vaccine injection are shown in the top two rows. Axial ^{18}FDG PET images of the right popliteal lymph node for all dose groups at baseline and 24 hr post Prime vaccine injection are shown in the bottom two rows. Orange arrows indicate right hindlimb signal enhancement in the MR images and indicate right popliteal lymph node ^{18}FDG uptake in the PET images at 24 hr post Prime vaccine injection.

Supplemental Figure S2

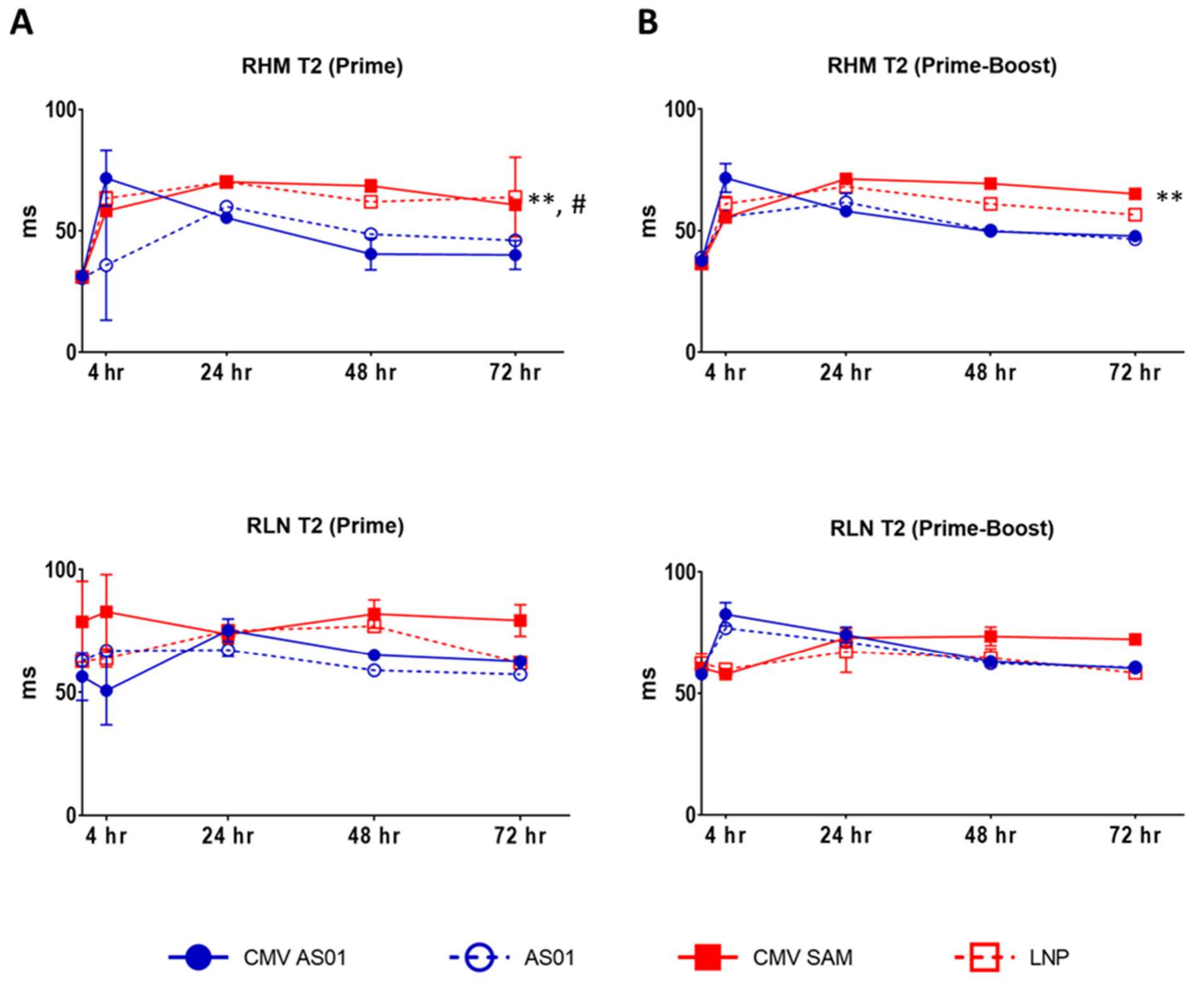

MRI T2 measurement in right hindlimb muscles (RHM) and right popliteal lymph node (RLN). T2 was measured following Prime (A) or Prime-Boost (B) vaccine injection in both RHM and RLN. Data are presented as mean ± SEM. **P<0.01 CMV SAM AUC vs CMV AS01 AUC; #P<0.05 AS01 AUC vs LNP AUC.

Supplemental Figure S3

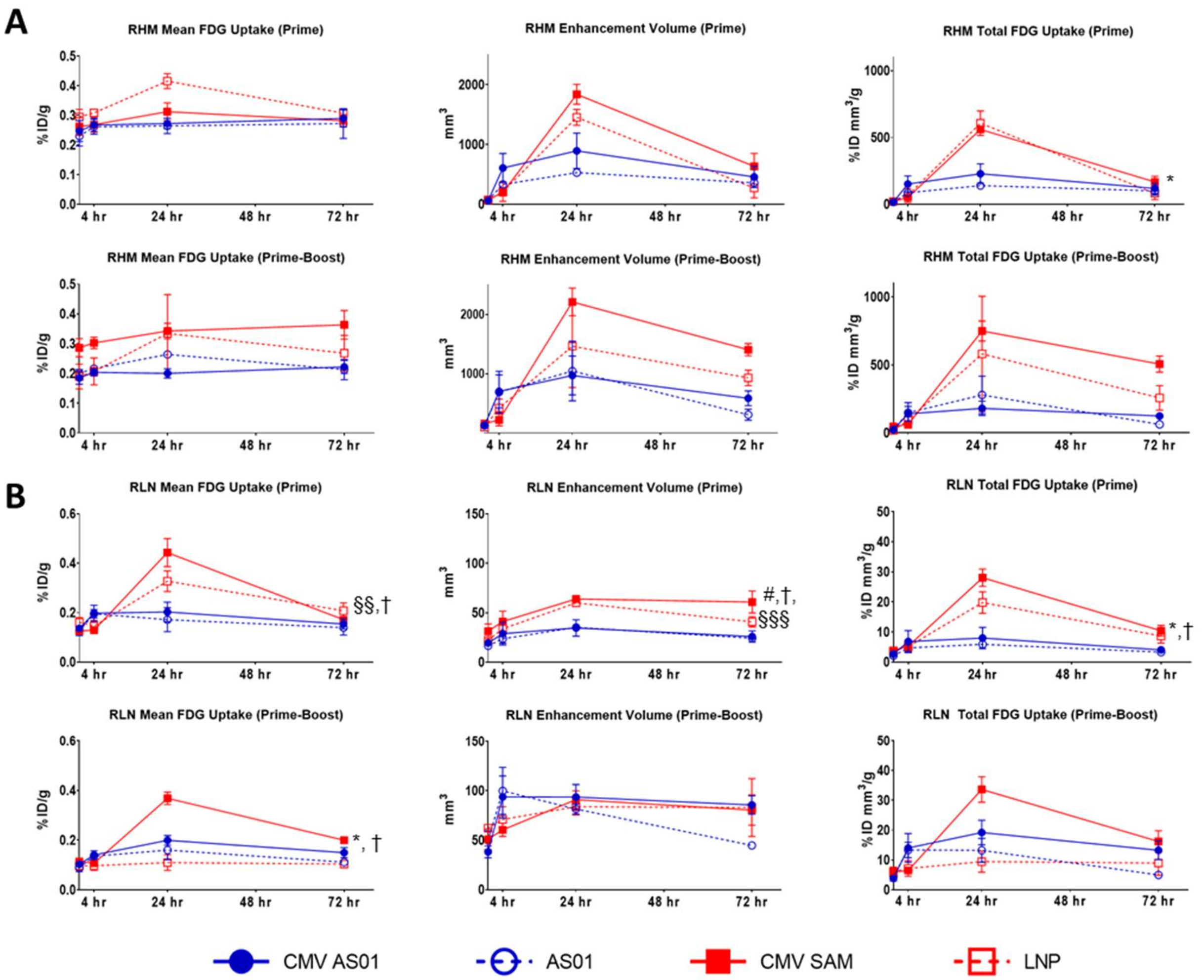

Quantitative PET data readouts in right hindlimb muscles (RHM) and right popliteal lymph node (RLN). ^{18}FDG PET derived endpoints including mean FDG uptake and FDG enhancement volume were used to calculate total FDG uptake in RHM (A) and in RLN (B) following Prime or Prime-Boost vaccine injection. Data are presented as mean ± SEM. *P<0.05 CMV SAM AUC vs CMV AS01 AUC; †P<0.05 CMV SAM maximum vs CMV AS01 maximum; $^{\#}$P<0.05 CMV AS01 maximum (Prime) vs CMV AS01 maximum (Prime-Boost); §§P<0.01 LNP AUC (Prime) vs LNP AUC (Prime-Boost); §§§P<0.001 CMV AS01 AUC (Prime) vs CMV AS01 AUC (Prime-Boost).

Supplemental Figure S4

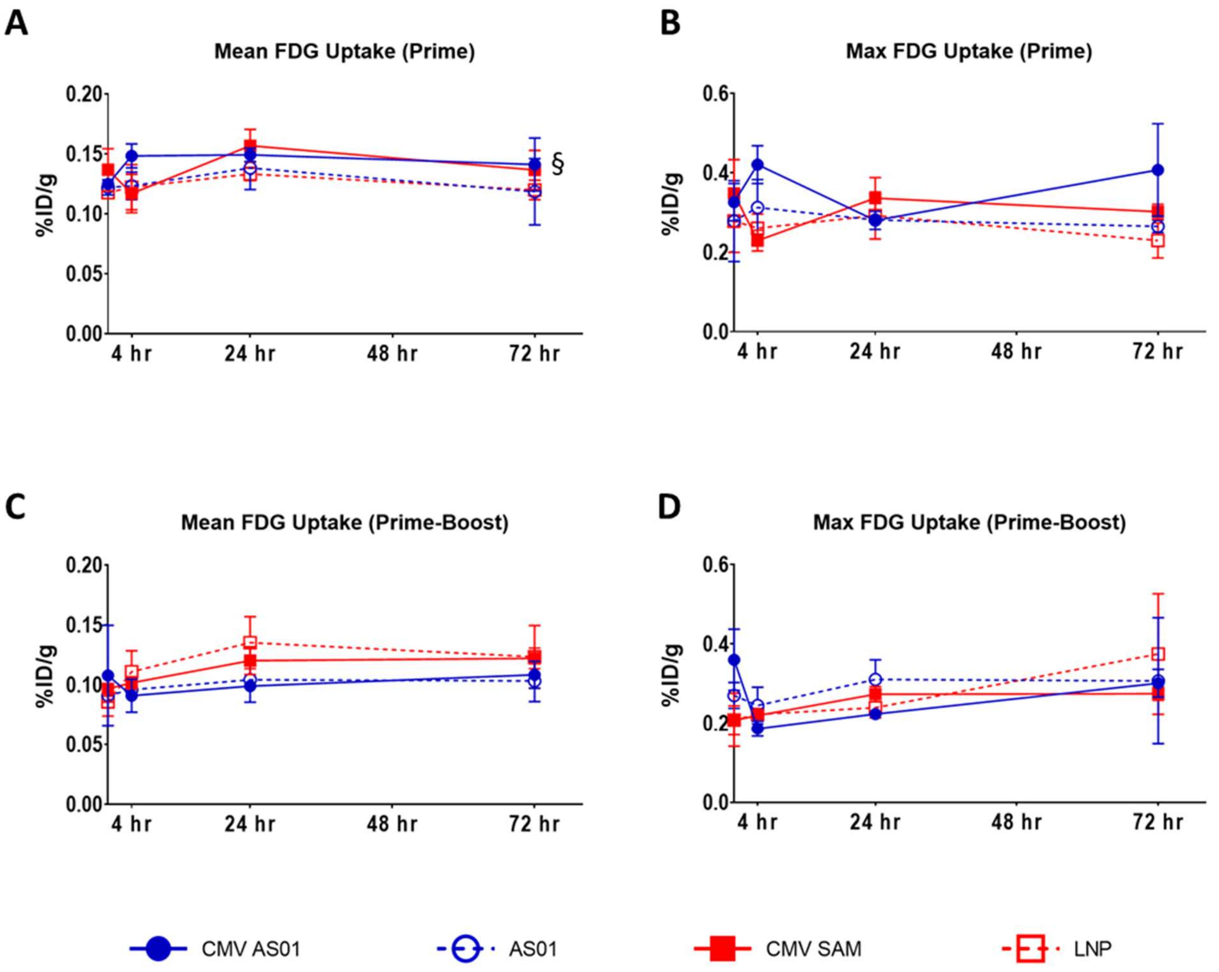

Spleen mean and maximum ¹⁸FDG uptake. Spleen mean (A) and max (B) FDG uptake was measured following Prime vaccine injection. Spleen mean (C) and max (D) FDG uptake was measured following Prime-Boost vaccine injection. Data are presented as mean ± SEM. $^{§}P<0.05$ CMV AS01 AUC (Prime) vs CMV AS01 AUC (Prime-Boost).

Supplemental Figure S5

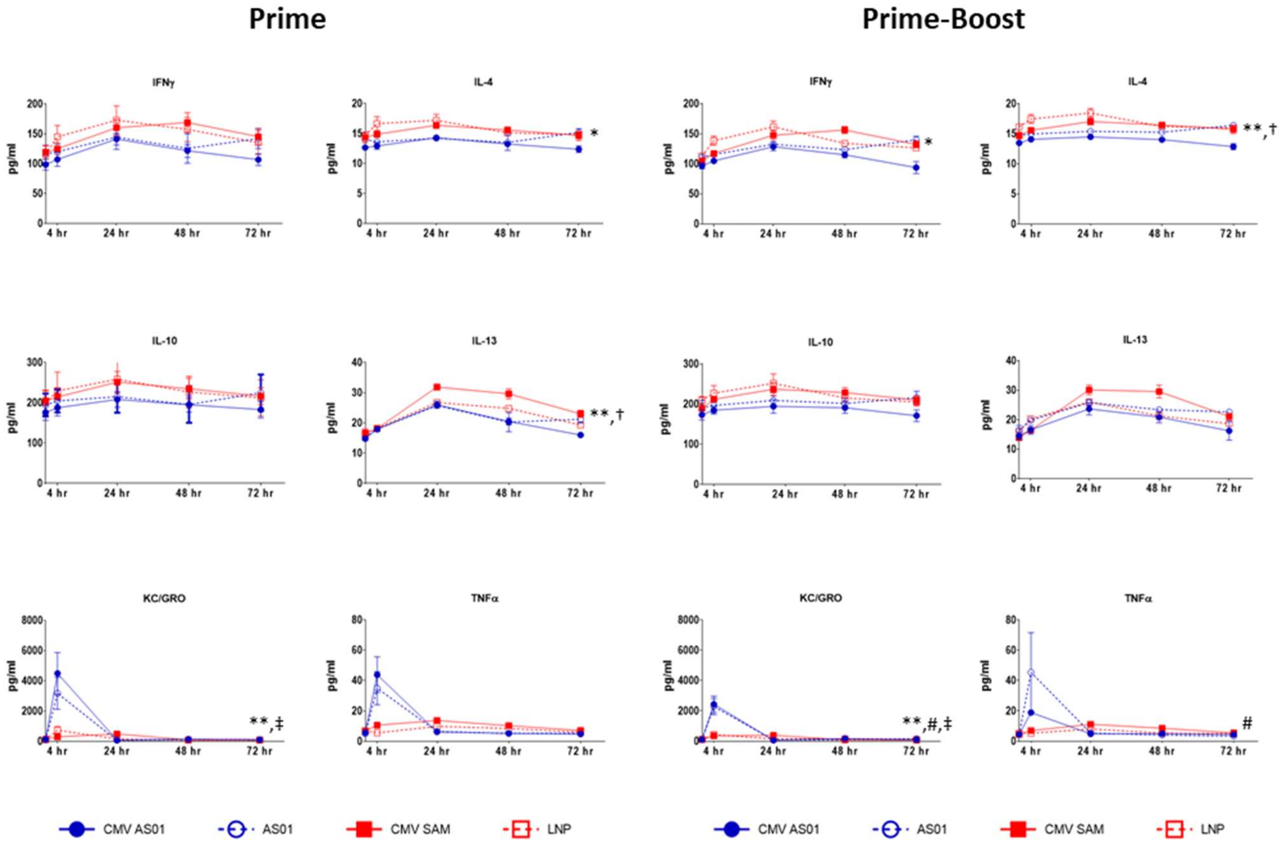

Plasma cytokine response. Plasma cytokines (IFNγ, IL-4, IL-10, IL-13, KC/GRO, and TNFα) were temporally assessed following Prime and Prime-Boost vaccine injection. Data are presented as mean ± SEM. *P<0.05 CMV SAM AUC vs CMV AS01 AUC; **P<0.01 CMV SAM AUC vs CMV AS01 AUC; †P<0.05 CMV SAM maximum vs CMV AS01 maximum; ‡P<0.01 CMV SAM maximum vs CMV AS01 maximum; #P<0.05 AS01 AUC vs LNP AUC.

Supplemental Figure S6

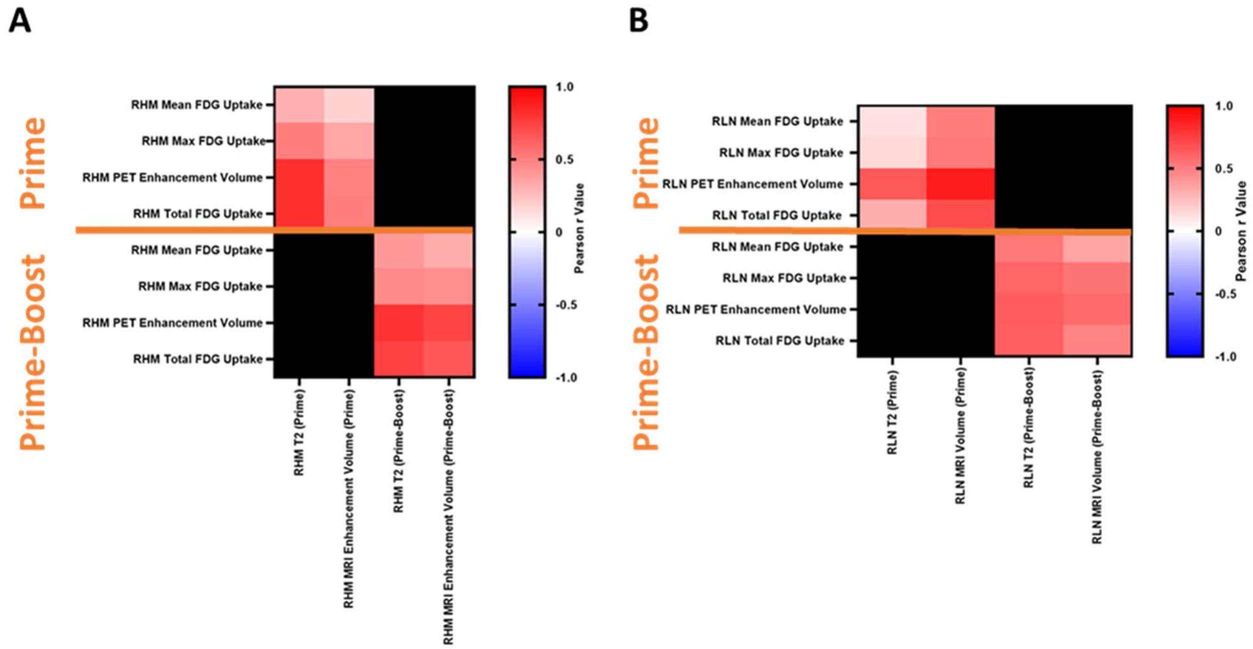

Correlation assessment between MRI and PET imaging endpoints. Heat maps of Pearson correlations between MRI endpoints in right hindlimb muscles (RHM T2 and RHM signal enhancement volume) and RHM ^{18}FDG PET endpoints (mean FDG uptake, max FDG uptake, FDG enhancement volume, and total FDG uptake) following Prime and Prime-Boost vaccine injection (A). Heat maps of Pearson correlations between MRI endpoints in right popliteal lymph node (RLN T2 and RLN signal enhancement volume) and RLN ^{18}FDG PET endpoints (mean FDG uptake, max FDG uptake, FDG enhancement volume, and total FDG uptake) following Prime and Prime-Boost vaccine injection (B).

Supplemental Figure S7

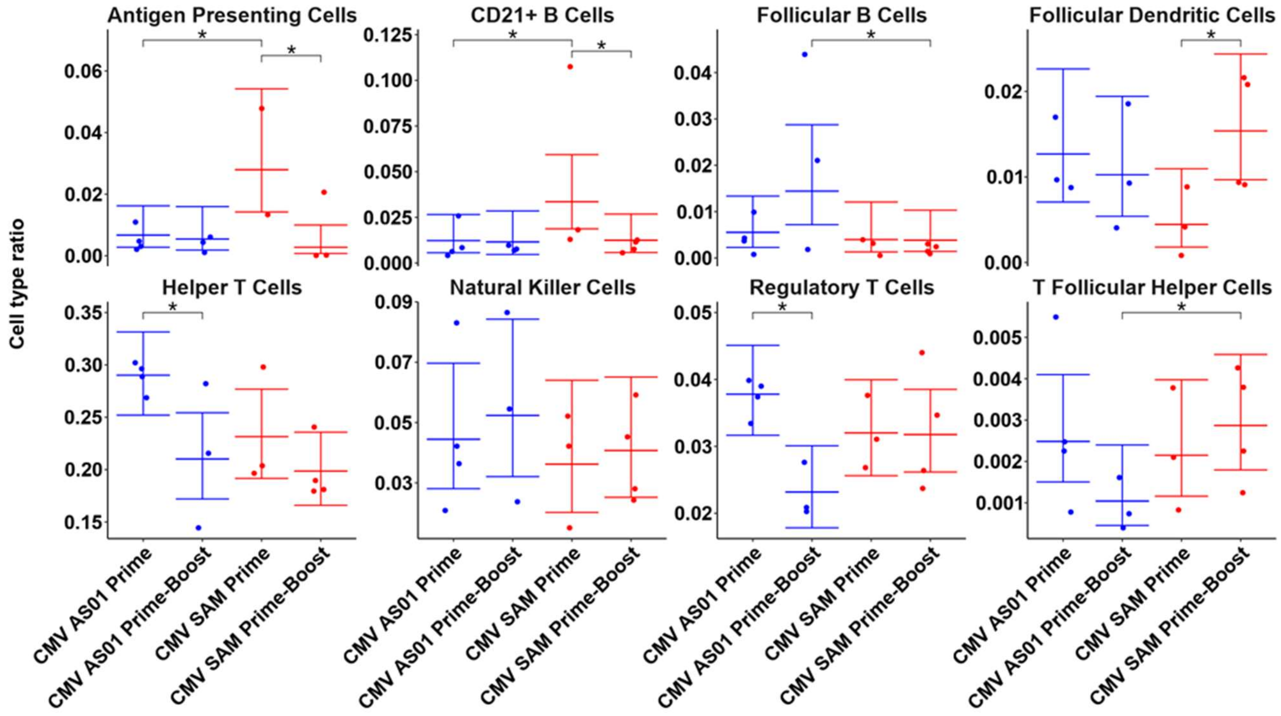

Immune cell type ratio derived from IMC data. Points represent the cell type ratio for an individual animal. Cell type ratios were modelled with a beta regression specifying logit link. For any ratio of zero, a pseudo count of 10^{-5} was added. The data are presented as mean and the 95% CI. *P<0.05.

Supplemental Figure S8

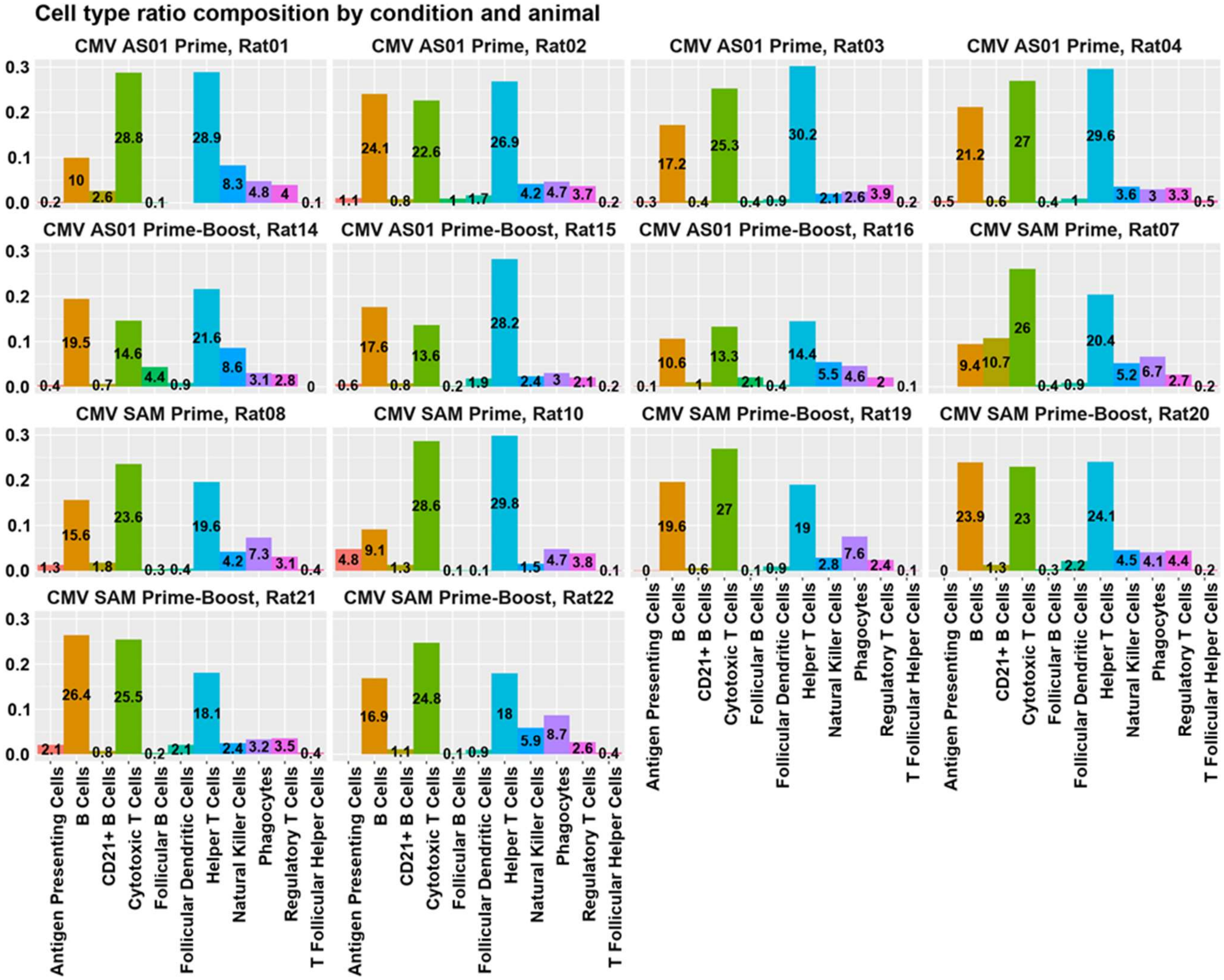

Individual animal cell type composition. Cell type ratios are presented for all animals in Prime and Prime-Boost cohorts in which IMC data was successfully obtained. X-axis provides the name of the cell type and y-axis – the proportion of the cell type observed. The numbers on the graph state the percentage of the cell type of interest. A blank reflects no cells of this particular cell type observed, and zero reflects < 0.1% of this cell type present in the sample.

Supplemental Figure S9

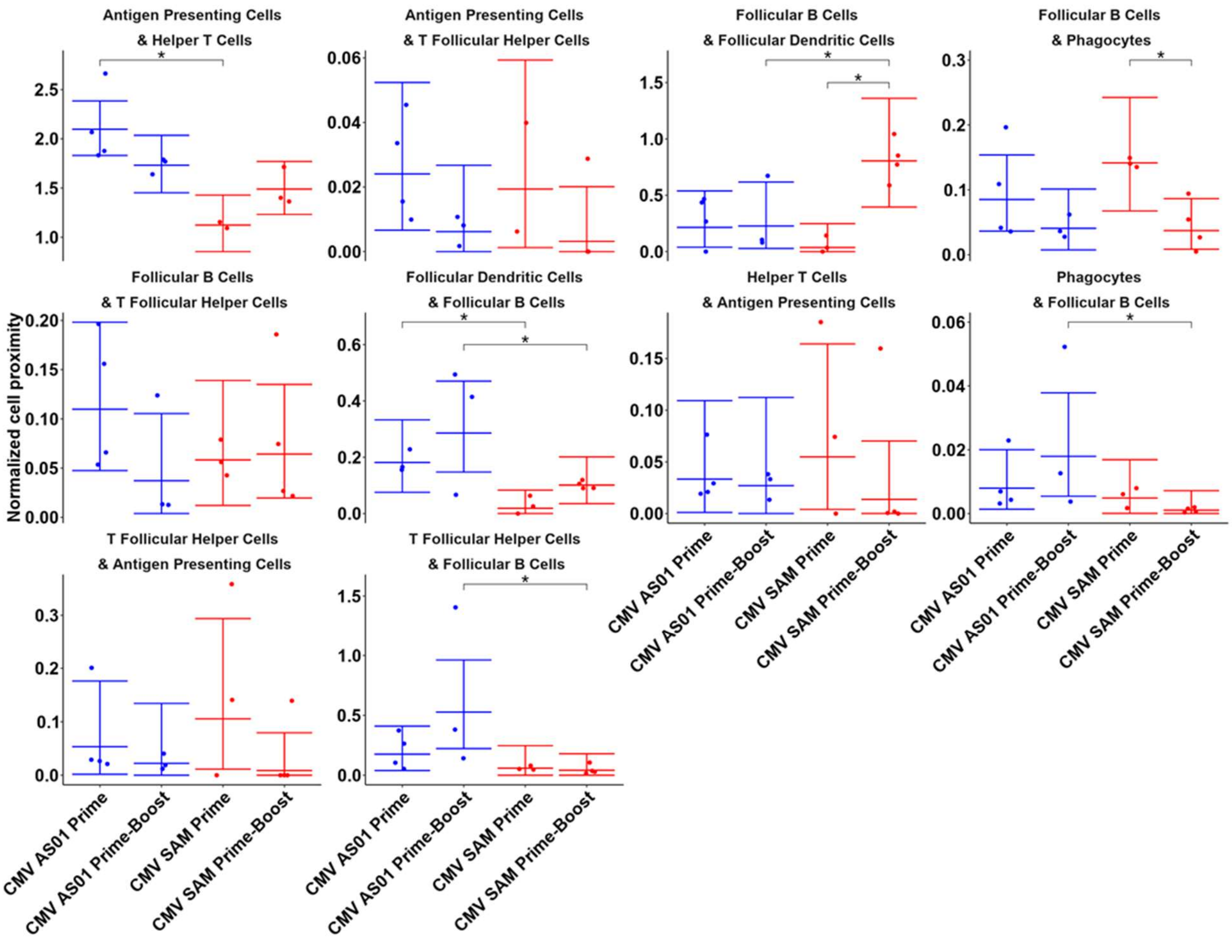

Immune cell type proximity analysis derived from IMC data. Points represent the cell type proximity for an individual animal, defined as the average number of cells of the second listed cell type that are within a short distance from each cell of the first listed type. Cell type proximities were square-root transformed and modelled with a linear regression. The data are presented as mean and the 95% CI. *P<0.05.

Supplemental Figure S10

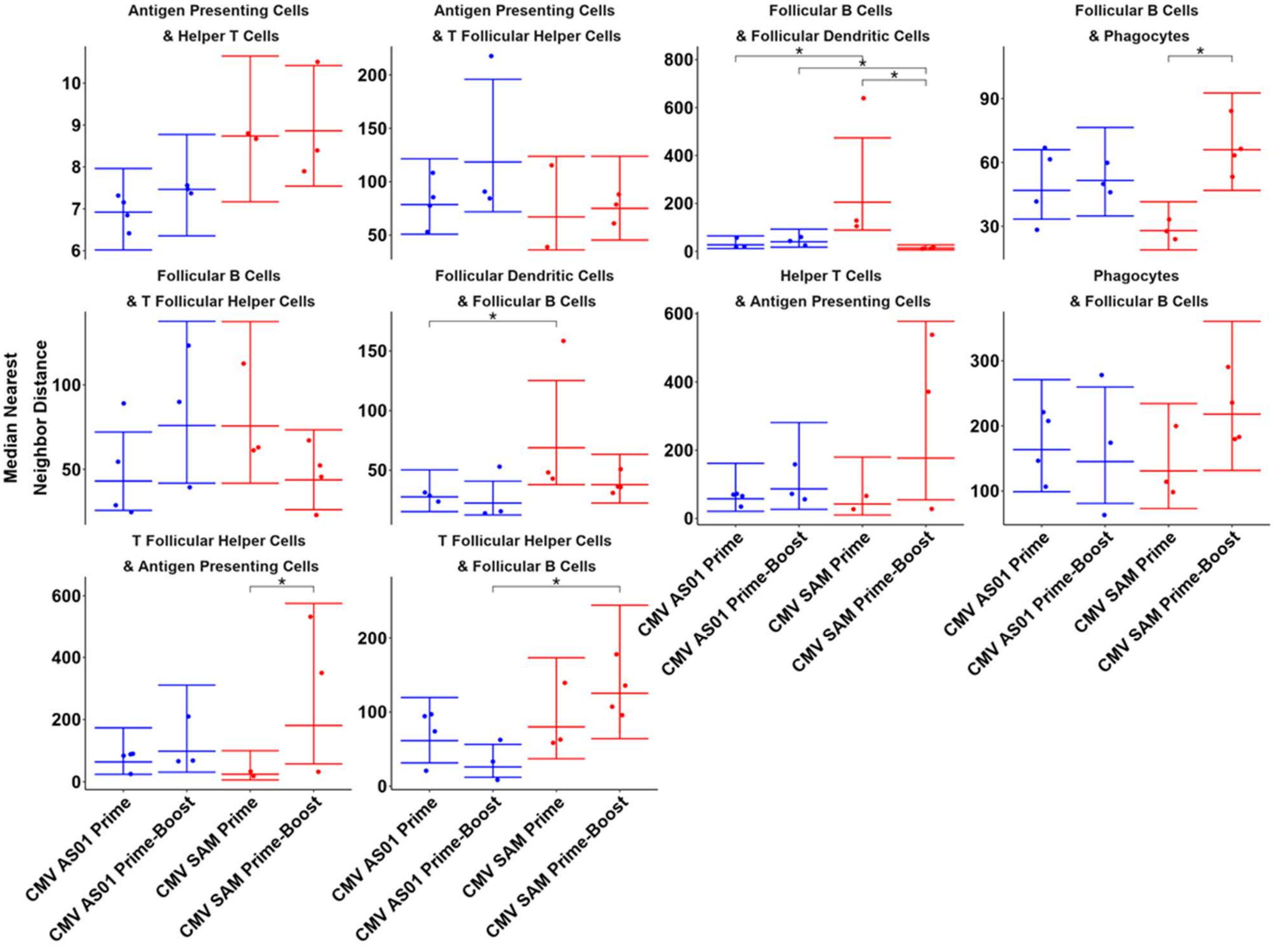

Immune cell type nearest neighbor analysis derived from IMC data. Points represent the cell type nearest neighbor distance for an individual animal, defined as the median distance between each cell of the first listed type and its closest cell of the second listed type. Nearest neighbor distances were log-root transformed and modelled with a linear regression. The data are presented as mean and the 95% CI. *P<0.05.

Supplemental Table S1

Antibodies used for Imaging Mass Cytometry

Isotope	Antigen	Clone	Final Conc. [µg/mL]	Supplier
149Sm	MHCII	MRC OX-6	1.25	Abcam
152Sm	CD8a	OX-8	2.5	Abcam
153Eu	CD21	SP186	0.625	Abcam
156Gd	CD4	CAL4	2.5	Abcam
162Dy	CD20	SP32	2.5	Abcam
164Dy	CD68	ED1	2.5	Abcam
165Ho	FoxP3	EPR22102-37	2.5	Abcam
166Er	NCR1	EPR23097-35	0.625	Abcam
170Er	CD3	SP7	5	Abcam
171Yb	Bcl6	EPR11410-43	2.5	Abcam

Supplemental Table S2

Correlation analysis between IMC results and MRI/PET imaging endpoints or serum antibody titers

IMC Readout	MRI/PET Readout/Serum Ab titer	Correlation Coefficient	p-value	Sample number
Median Nearest Neighbor Follicular Dendritic Cells to Follicular B Cells	RLN T2	0.808	0.00009	17
Ratio Antigen Presenting Cells	RLN T2	0.722	0.00159	16
Median Nearest Neighbor Antigen Presenting Cells to Helper T Cells	RHM Enhancement volume	0.704	0.00232	16
Ratio Phagocytes	RHM Enhancement volume	0.700	0.00121	18
Ratio Cytotoxic T Cells	gB Ab	-0.918	0.00356	7

Supplemental Radiomics Description:

Interquartile range is the difference between 75th percentile and 25th percentile.

Energy is a measure of the magnitude of voxel values in an image. A larger value implies a greater sum of the squares of these values.

Dependence Non Uniformity Measures the similarity of dependence throughout the image, with a lower value indicating more homogeneity among dependencies in the image
